# Controlled atom transfer radical polymerization of MMA onto the surface of high-density functionalized graphene oxide

**DOI:** 10.1186/1556-276X-9-345

**Published:** 2014-07-10

**Authors:** Mukesh Kumar, Jin Suk Chung, Seung Hyun Hur

**Affiliations:** 1 School of Chemical Engineering, University of Ulsan, Daehakro 93, Ulsan, Namgu 680-749, Korea

**Keywords:** Graphene oxide, PMMA nanocomposites, Controlled radical polymerization, ATRP

## Abstract

We report on the grafting of poly(methyl methacrylate) (PMMA) onto the surface of high-density functionalized graphene oxides (GO) through controlled radical polymerization (CRP). To increase the density of surface grafting, GO was first diazotized (DGO), followed by esterification with 2-bromoisobutyryl bromide, which resulted in an atom transfer radical polymerization (ATRP) initiator-functionalized DGO-Br. The functionalized DGO-Br was characterized by X-ray photoelectron spectroscopy (XPS), Raman, and XRD patterns. PMMA chains were then grafted onto the DGO-Br surface through a ‘grafting from’ technique using ATRP. Gel permeation chromatography (GPC) results revealed that polymerization of methyl methacrylate (MMA) follows CRP. Thermal studies show that the resulting graphene-PMMA nanocomposites have higher thermal stability and glass transition temperatures (*T*_g_) than those of pristine PMMA.

## Background

The discovery of two-dimensional (2D) sp^2^ hybridized graphene sheets by Novoselov [1] in 2004 has received much attention due to their extraordinary electrical, thermal, and mechanical properties [1-5]. Due to its high surface-area-to-volume ratio, graphene has been effectively used in the synthesis of polymer nanocomposites which exhibit enhanced physical and chemical properties over individual components [6]. The functionalization of graphene has received much attention in recent years as a way to improve interfacial interactions with other components, including organic and inorganic polymers, as the key to maximizing the end properties of the resulting graphene-polymer nanocomposites is controlling the dispersion of graphene within the matrix of the main components [7-9]. Moreover, the functional groups may not only improve the miscibility of graphene in organic solvents but also may provide nucleation sites for efficient *in situ* grafting of polymeric chains onto the graphene surface, which results in further improvements in mechanical and thermal properties [10].

Efforts to enhance the end properties of graphene-polymer nanocomposites using surface polymerization through *in situ* ‘grafting to’ and ‘grafting from’ techniques have been reported [11,12]. *In situ* polymerization offers the ability to control the polymer architecture and final morphology of the resulting composites. Ramanathan et al. reported an extraordinary shift in glass transition temperature (*T*_g_), modulus, ultimate strength, and thermal stability for poly(acrylonitrile) and poly(methyl methacrylate) using very low levels of functionalized graphene sheets [13]. *In situ* emulsion polymerization of methyl methacrylate (MMA) was carried out by Kuila et al. using graphene as a reinforcing filer, which also enhanced the storage moduli, *T*_g_, and thermal stability of the resulting nanocomposites [14].

Living ionic polymerization has been widely used to produce homo- and block copolymers with well-defined architectures, controlled molecular weights, and narrow polydispersity index (PDI). However, the industrial applications of ionic polymerization are limited due to the need for rigorous polymerization conditions, such as highly purified monomers and solvents. In addition, living ionic polymerization can only be used to polymerize hydrocarbon monomers and the polar monomer due to unwanted side reactions. Atom transfer radical polymerization (ATRP) is an alternative polymerization technique to improve polymer architectures under simple polymerization conditions in the presence of hydrophilic organic/inorganic fillers such as layered silicates and graphene oxide (GO) [15,16]. The direct growth of thermoresponsive poly-2-(dimethylamino)ethyl methacrylate polymer brushes with narrow PDI through surface-initiated highly controlled ATRP from initiator functionalized graphene sheets has been reported, where the polymers were grown directly from a graphene surface with covalent functionalization to tune the physical, thermal, and electrical properties [17]. Pyrene-based functionalized graphene has been used for reversible addition fragmentation chain transfer (RAFT) polymerization of dimethyl aminoethyl acrylate, acrylic acid, and styrene in order to avoid graphene aggregation [18]. The efficient functionalization through diazotization of graphene for ATRP of styrene results in high-performance polymer-graphene nanocomposites with increased tensile strength, *T*_g_ and Young's modulus [19]. Covalently bounded polystyrene polymer chains have been systematically tuned using ATRP on single-layer graphene nanosheets by Fang et al. [20]. High-density grafted polymer-graphene nanocomposites exhibit an appreciable increase in *T*_g_ compared with low-density grafted samples.

In this study, we focused on the functionalization of GO and highs-density grafting of poly(methyl methacrylate) (PMMA) chains onto its surface through an *in situ* ‘grafting from’ technique using ATRP. Quaternization and esterification after diazotization were carried out to increase the number of anchoring sites for ATRP initiators for increased grafting of polymer chains on the GO surface. ATRP of MMA was carried out using GO with ATRP initiators on the surface, cupric bromide (CuBr, catalyst), and N,N,N′,N″,N″-pentamethyldiethylenetriamine (PMDETA, ligand) at ambient temperature. The resulting graphene-PMMA nanocomposites showed higher thermal stability and higher glass transition temperatures (T_*g*_) than pristine PMMA polymers.

## Methods

Acid-treated natural expandable graphite (grade 1721) was purchased from Asbury Carbons, Asbury, NJ, USA. Concentrated sulfuric acid (H_2_SO_4_), potassium permanganate (KMnO_4_), sodium nitrate (NaNO_3_), sodium nitrite (NaNO_2_), sodium carbonate (Na_2_CO_3_), hydrochloric acid (HCl, 35%), hydrogen peroxide (H_2_O_2_, 30 wt.%), N,N′-dimethylformamide (DMF), MMA, 2-chloroethanol, *p*-aminobenzoic acid, and 2,2',2"-trihydroxy-triethylamine (triethanolamine) were purchased from Daejung Reagents & Chemicals, Ulsan, Korea. Cuprous bromide (CuBr), N,N,N′,N″,N″-PMDETA and polystyrene standards for gel permeation chromatography (GPC) were purchased from Sigma-Aldrich, St. Louis, MO, USA and were used as received without further purification. The stabilizing agent was removed from commercial MMA by washing three times with sodium hydroxide (NaOH), followed by vacuum distillation; the middle portion was stored at 0°C to 4°C until use. DMF was stirred with anhydrous calcium hydride (CaH_2_) and then distilled before use.

### Preparation of DGO-Br

The preparation steps of GO, diazotized GO (DGO-COOH), tetrakis(2-hydroxyethyl) ammonium chloride (THAC), DGO-COO^−^Na^+^, and DGO-OH have been reported in our previous paper [21]. DGO-Br was prepared by the esterification of α-bromoisobutyryl bromide with DGO-OH. In brief, 2 g of DGO-OH powder was dispersed in distilled dry DMF using sonication, and 1 mL of triethyl amine was added to the suspension under a nitrogen atmosphere. The α-bromoisobutyryl bromide was added slowly to the above suspension at 0°C using a gas-tight syringe. The reaction mixture was stirred at the same temperature for 6 h and then increased to 25°C and stirred for 12 h. The resulting suspension (DGO-Br) was centrifuged and washed repeatedly with acetone and methanol and dried at 65°C in a vacuum oven.

### Polymerization of MMA on the surface of DGO-Br

The ATRP of MMA was carried out using the prepared DGO-Br. In a typical procedure, 30 mg of DGO-Br was dissolved in 5 mL of distilled dry DMF and was homogenously dispersed by ultrasonication for 30 min before starting polymerization. Next, 15 mg of CuBr, 15 μL of PMDETA catalyst, and 5 mL of MMA were added successively. The reaction mixture was then degassed three times and vacuum-sealed with a septum, followed by nitrogen purging for 30 min to evacuate the residual oxygen. The mixture was then placed in a thermo-stated oil bath at 80°C for the designated period of time. Polymerization was stopped by quenching the polymerization tube in ice cold water. The resulting solution was poured into a petri dish to evaporate the excess solvent. The polymerization yields were calculated gravimetrically.

### Detachment of the polymer chains from the GO surface

To determine the molecular weight by GPC, polymeric chains were detached from the surface of DGO-Br through a reverse cation exchange process. In brief, the resulting graphene-PMMA nanocomposite (0.5 g) was dissolved in 50 mL of tetrahydrofuran (THF), and lithium chloride (0.05 g) was added to the reaction mixture. The solution was refluxed for 24 h and filtered through Celite (Sigma-Aldrich). The free polymer was recovered by adding the filtrate into methanol and was then filtered and dried in a vacuum oven.

### Characterization

Raman spectra were recorded using a confocal Raman spectrometer (Alpha300S, WITec, Ulm, Germany) with a 633-nm wavelength incident laser light. The crystallographic structures of the materials were determined by a wide-angle X-ray diffraction (WAXRD) system (Rigaku RU-200 diffractometer, Shibuya-ku, Japan) equipped with a Ni-filtered Cu Kα source (40 kV, 100 mA, *λ* = 0.15418 nm). Bragg's equation (*nλ* = 2*d*sin*θ*) was used to calculate the *d* spacing between the layers. X-ray photoelectron spectroscopy (XPS) was performed to determine the oxidation status of carbon using a Thermo Fisher X-ray photoelectron spectrophotometer (Waltham, MA, USA) employing an Al Kα X-ray source (1,486.6 eV). Thermogravimetric analysis (TGA) was performed to analyze the thermal behavior of the samples using a TGA analyzer (Q50, TA Instruments, New Castle, DE, USA) with a 10°C min^−1^ heating rate in a nitrogen atmosphere. Number of average molecular weight (${\bar{M}}_{n}$), weight average molecular weight (${\bar{M}}_{w}$), and molecular weight distribution (MWD) were determined using a Waters GPC instrument (Milford, MA, USA) equipped with a 2414 differential refractive index detector and two (HR 2 and HR 4) Waters μ-Styragel columns. Spectral grade THF was used as an eluent at a flow rate of 1.0 ml min^−1^, and the molecular weight calibrations were carried out using polystyrene standards.

## Results and discussion

**Figure 1:**
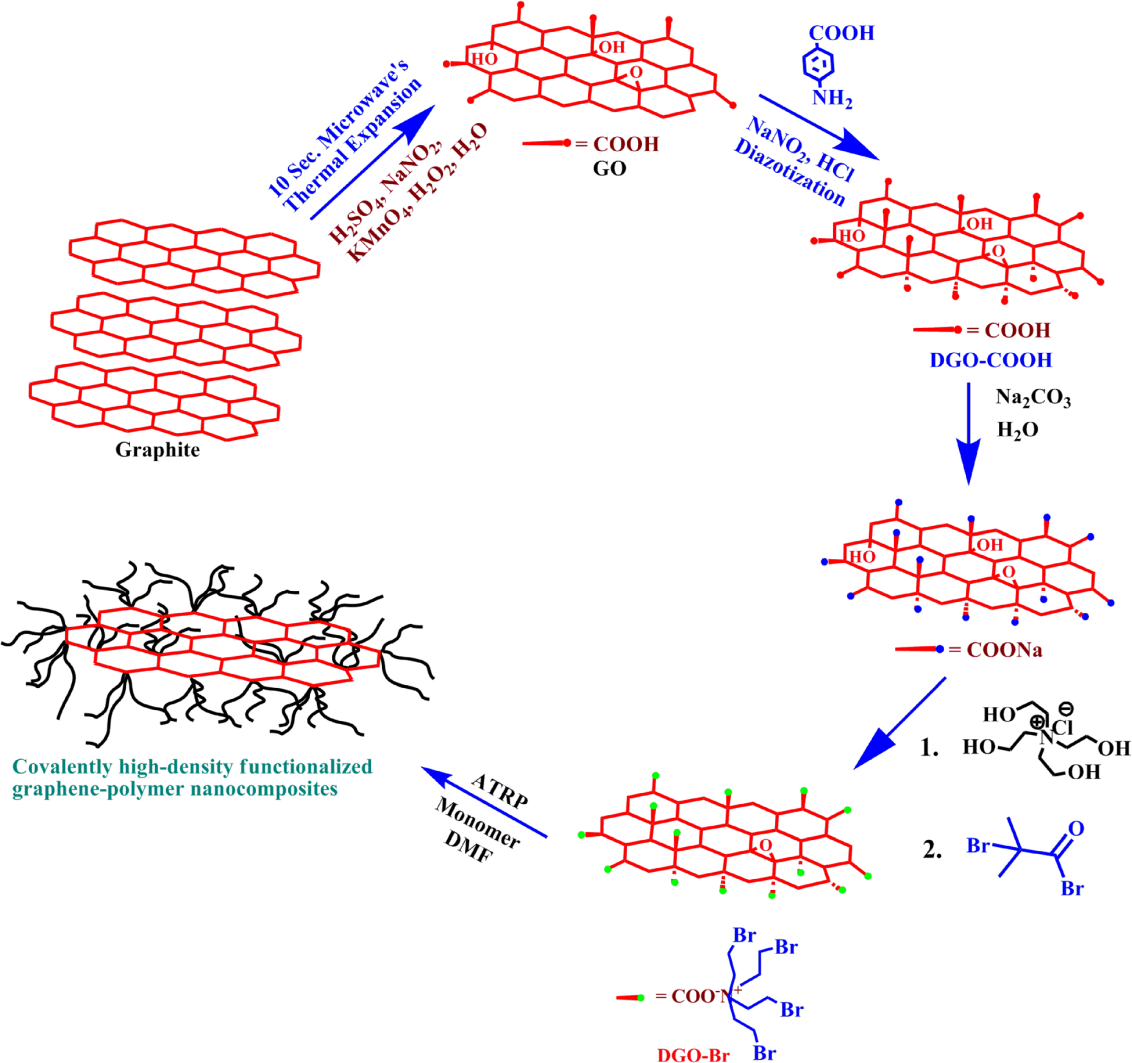
Schematic representation of the synthetic procedures of the graphene-polymer nanocomposites.

**Figure 2:**
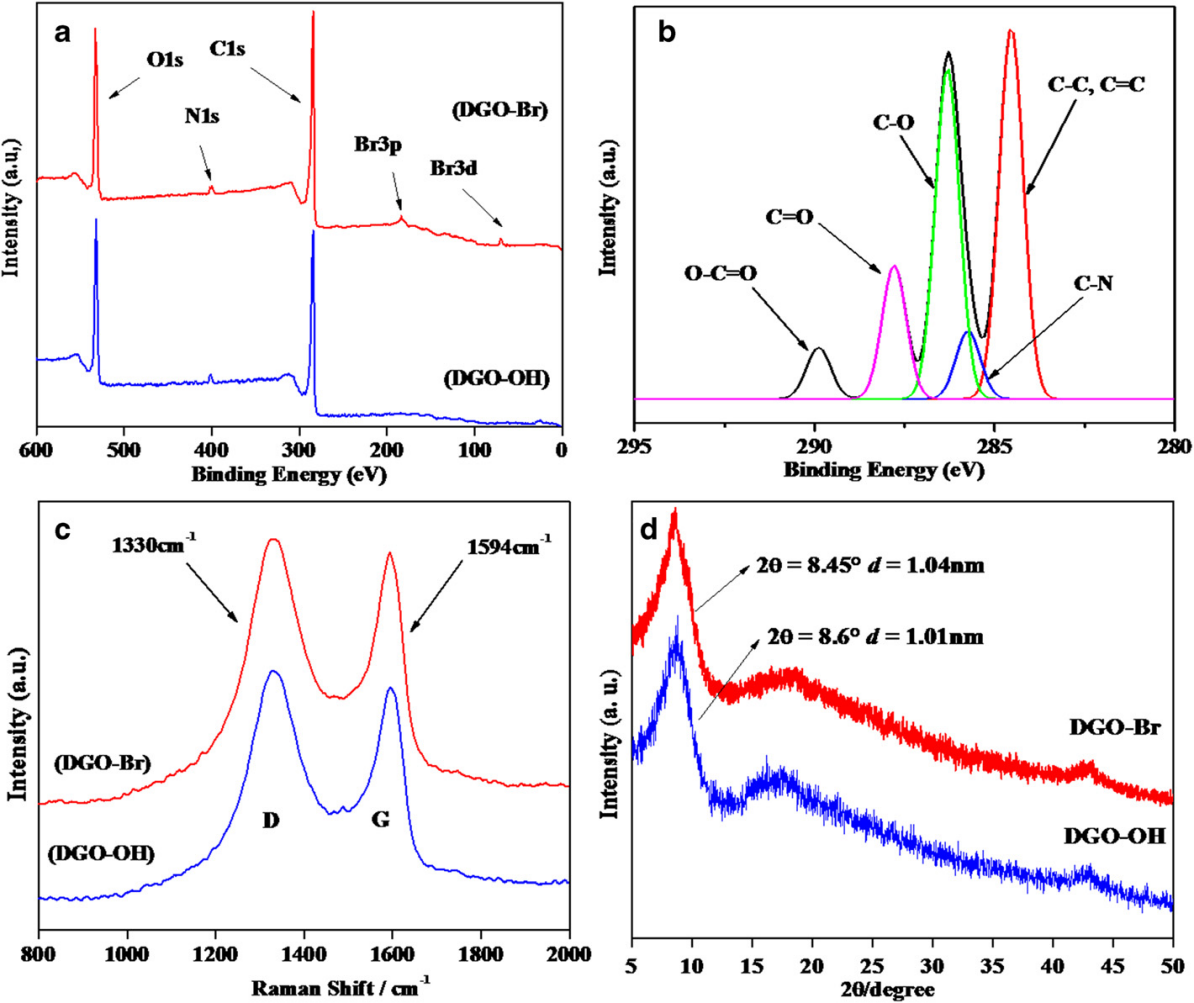
**XPS survey data, C1s core level data, Raman spectra, and XRD pattern.** XPS survey data of **(a)** (i) DGO-OH, (ii) DGO-Br; C1s core level data of **(b)** DGO-Br; Raman spectra of **(c)** (i) DGO-OH, (ii) DGO-Br; and XRD pattern of **(d)** (i) DGO-OH, (ii) DGO-Br.

In general, good interaction between fillers and polymers leads to significant improvements in the properties of the resulting final products. To increase the interfacial interactions between GO and the polymers, the GO was first diazotized with *p*-aminobenzoic acid to obtain DGO-COOH, followed by a quaternization reaction with THAC and an esterification reaction with α-bromoisobutyryl bromide, which resulted in a tertiary bromine-terminated DGO-Br for efficient ATRP, as shown in Figure 1. Detailed characterizations of GO, DGO-COOH, and DGO-OH through FT-IR, Raman, XPS, XRD, and TGA have been reported in our previous paper [21]. In addition, XPS was used to investigate the changes in the functional groups of DGO-OH and DGO-Br, as shown in Figure 2a. Two intense peaks at 285 and 532 eV can be attributed to C1s and O1s, respectively [22]. The new peak of N1s at 399 to 400 eV was observed by diazotization. The C/O ratios of the functionalized DGO-OH and DGO-Br were 2.5 and 2.65, respectively, which can be correlated with dehydration during the esterification of DGO-OH to DGO-Br. The deconvoluted C1s XPS spectra of DGO-Br (Figure 2b) show several peaks at 284.5, 286.3, 287.9, and 289.7 eV originating from C-C, C-O, C = O, and O-C = O groups, respectively. In comparison to DGO-OH [21], the relative intensity of the C-C peak remains the same after esterification, but the intensity of the C = O and O-C = O peaks increased, which may be due to increased functionality.

Raman spectra of DGO-OH and DGO-Br are shown in Figure 2c. The G and D bands in the Raman spectra originate from the first-order scattering of E_2g_ phonons of sp^2^-bonded carbon atoms and with a breathing mode of j-point photons of A_1g_ symmetry of sp^3^-bonded carbon atoms of disordered graphene. The Raman spectrum of DGO-OH shows sp^2^-bonded carbon stretching related to the G band at 1,594 cm^−1^ and disordered, D band, sp^3^-bonded carbon atoms at 1,330 cm^−1^. The intensity ratio of the D and G bands (*I*_D_/*I*_G_) for DGO-OH and DGO-Br were 1.3 and 1.35, respectively. The slightly increased *I*_D_/*I*_G_ ratio may be due to increased functionalization after esterification. WAXRD patterns of DGO-OH and DGO-Br are shown in Figure 2d. The DGO-OH and DGO-Br exhibit peaks at 8.6° and 8.45°, indicating *d* spacings of 1.01 nm and 1.04 nm, respectively (based on Bragg's equation). The slightly increased *d* spacing of DGO-Br over DGO-OH can be also attributed to the esterification of DGO-OH with α-bromoisobutyryl bromide.

**Figure 3:**
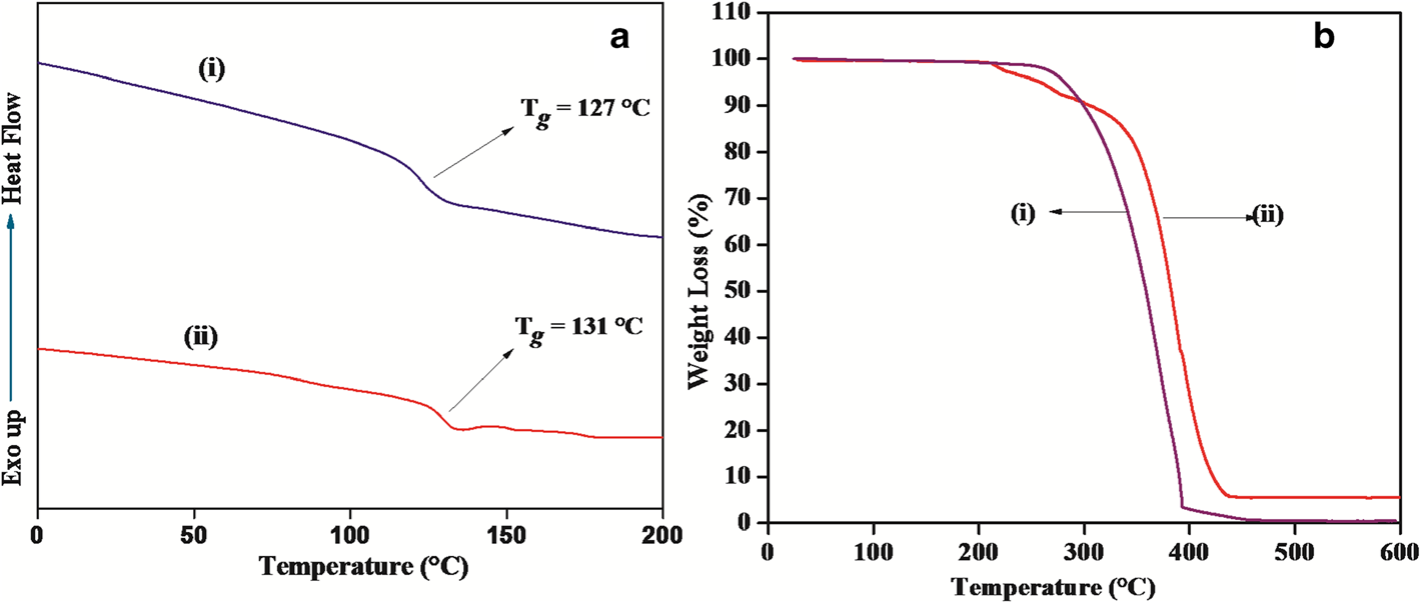
DSC results (a) of (i) PMMA and (ii) DGO-PMMA and TGA curves (b) of (i) PMMA and (ii) DGO-PMMA.

Thermal properties of the graphene-PMMA nanocomposites were compared with pristine PMMA by differential scanning calorimetry (DSC) and TGA. Figure 3 shows the DSC and TGA results for pristine PMMA and graphene-PMMA nanocomposite (GP-5) samples. For DSC (Figure 3a), the midpoints between the onset and offset points of the transition temperature were chosen as the *T*_g_ values. The graphene-PMMA nanocomposite showed a higher *T*_g_ than that of the pristine PMMA, which can be attributed to the interactions between GO and PMMA. The decomposition patterns for PMMA and GP-5 are shown in Figure 3b. About 15% of GP-5 nanocomposites decomposed between 130°C and 340°C, whereas pure PMMA decomposition started at 250°C. The initial decomposition of GP-5 may be due to the presence of additional labile functional groups after surface modification using quaternization followed by esterification onto the surface of GO [23]. On the other hand, the main decomposition of PMMA ends at 400°C, whereas that of the graphene-PMMA nanocomposite ends at 430°C. The difference in the thermal stability between pristine PMMA and GP-5 indicates that the presence of graphene layers improves the thermal properties of graphene-PMMA nanocomposites after *in situ* polymerization on the functionalized GO surface. The increased thermal stability of graphene-PMMA nanocomposites can be attributed to the attractive nature of graphene toward free radicals generated during decomposition as well as the tortuous path formation during the decomposition process [21,23].

### Controlled study of radical polymerization

**Figure 4:**
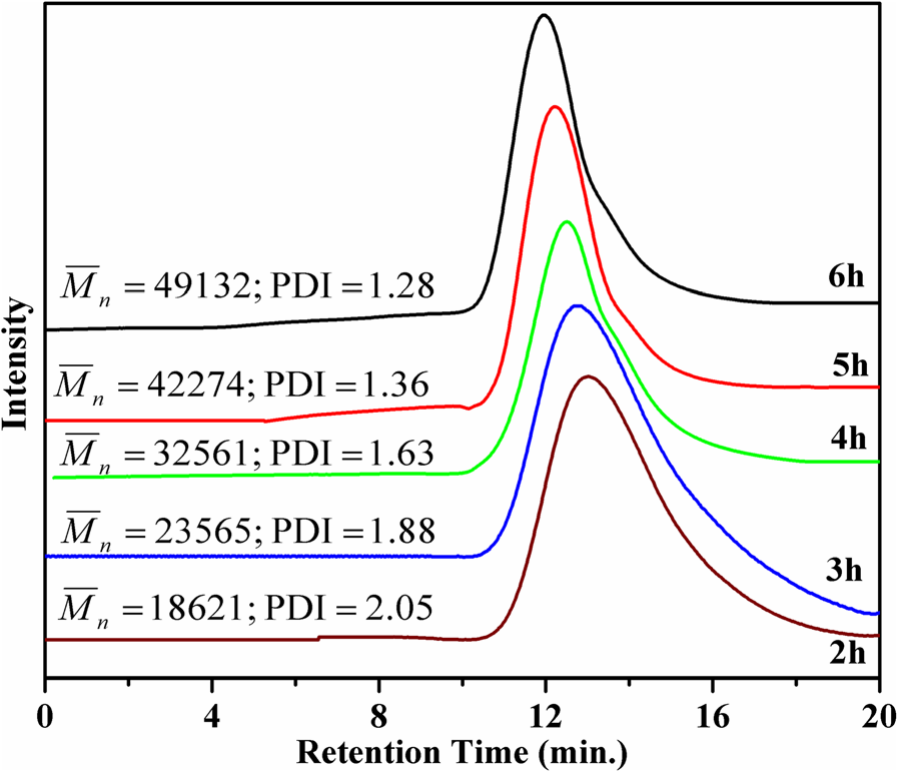
GPC curves of PMMA recovered from graphene-PMMA nanocomposites by reverse cation exchange.

**Figure 5:**
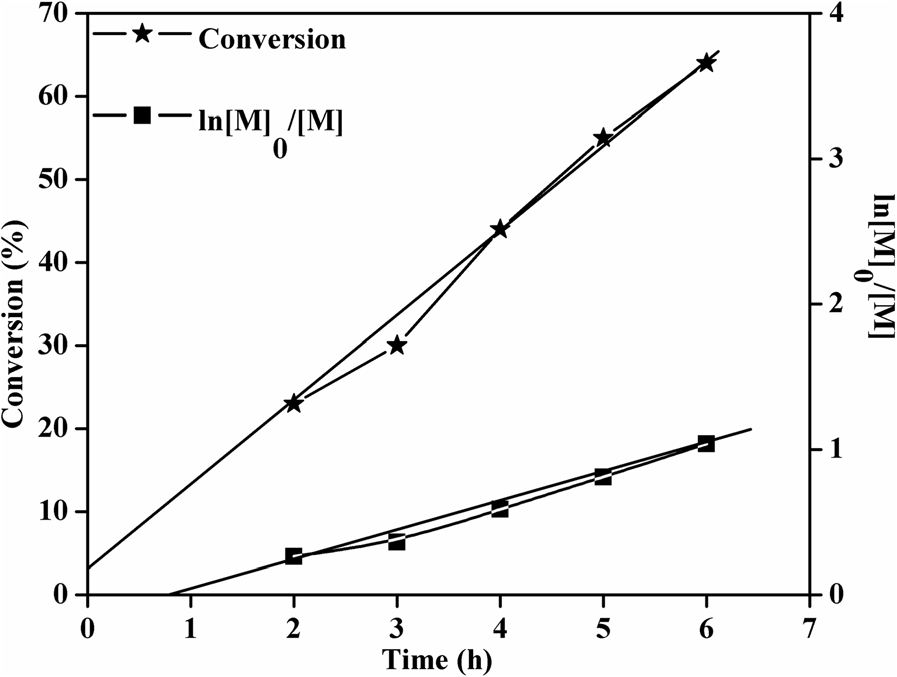
**Time vs. conversion and time vs. ln[M]**_**0**_**/[M] plots for the polymerization of MMA using DGO-Br.**

**Figure 6:**
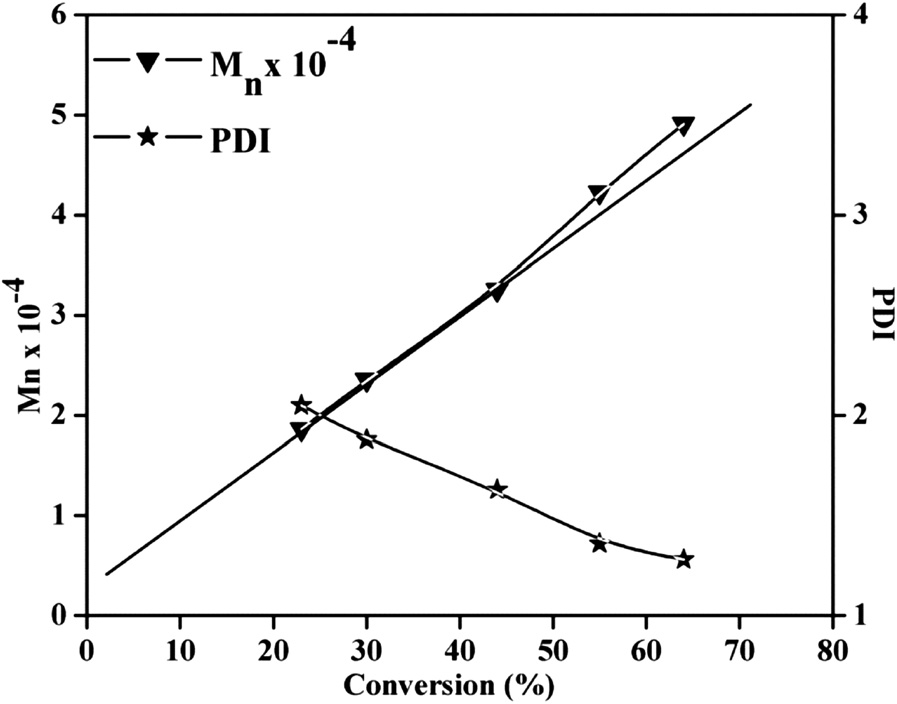
**Conversion vs.**${\bar{M}}_{n}$**and conversion vs. polydispersity index (PDI;**${\bar{M}}_{w}/{\bar{M}}_{n}$**) plots for the polymerization of MMA using DGO-Br.**

Polymerization of MMA was carried out through ATRP using multifunctional DGO-Br, and controlled radical polymerization (CRP) was studied using GPC. The detailed GPC results (${\bar{M}}_{n}$, ${\bar{M}}_{w}$, and MWD) are summarized in Table 1. As shown in Figure 4, as time increased, the GPC curves shifted from the lower molecular weight region to the higher molecular weight region due to the CRP mechanism. It is also interesting to note that the PDI values for PMMA become narrower with time, which also supports the CRP mechanism. Figure 5 shows the time vs. conversion and time vs. ln[M]_0_/[M] plots for MMA polymerization, where [M]_0_ and [M] represent the initial monomer concentration and the monomer concentration at time *t*, respectively. The linear relation between time vs. ln([M]_0_/[M]) shows that the concentration of propagating radicals is almost constant throughout the polymerization process. The small deviation in CRP results may be correlated with the induction period before starting the MMA polymerization, with similar results reported in previous literature [24,25]. As reported earlier, this induction period may account for the initial delay in reaching equilibrium between propagating radicals and dormant species. This delay may be responsible for the irreversible termination of some of the primary radicals in MMA polymerization, resulting in the deviations that are observed in the time vs. ln([M]_0_/[M]) plot. Further CRP was confirmed by plotting conversion against the number of average molecular weight (${\bar{M}}_{n}$), as shown in Figure 6. A linear increase in molecular weight was observed with increasing monomer conversion, which confirms that the polymerization of MMA on the DGO-Br proceeds through the CRP mechanism. The deviation of the conversion vs. ${\bar{M}}_{n}$ plot is also correlated with slow initiation. These plots show that MMA polymerization undergoes an induction period with slow initiation, as reported previously [25].

**Table 1:** Polymerization of MMA using TPEBMP at 80°C in DMF using DGO-Br

**Code**	**Time (h)**	^**a**^**Conversion (%)**	**GPC results**		
			${\bar{M}}_{w}\times 10^{‒4}$	${\bar{M}}_{n}\times 10^{‒4}$	${\bar{M}}_{w}/{\bar{M}}_{n}$
GP-1	2	23	3.8173	1.8621	2.05
GP-2	3	30	4.4302	2.3565	1.88
GP-3	4	44	5.3074	3.2561	1.63
GP-4	5	55	5.7492	4.2274	1.36
GP-5	6	64	6.2888	4.9132	1.28

^a^Conversion was determined gravimetrically.

## Conclusions

ATRP initiator-attached high-density functionalized graphene oxide (DGO-Br) was prepared and used for MMA polymerization, resulting in graphene-PMMA nanocomposites through controlled radical polymerization. DSC and TGA studies show that the graphene-PMMA nanocomposites exhibited higher T_*g*_ and higher thermal stability compared to pristine PMMA polymers. GPC results confirmed the presence of a controlled radical polymerization mechanism using functionalized DGO-Br. We believe that high-density functionalized GO can be used to develop graphene-polymer nanocomposites with enhanced properties.

## Competing interests

The authors declare that they have no competing interests.

## Authors' contributions

MK has designed all the conducted experiments and characterization for final publication. JSC and SHH have approved the final manuscript. All authors read and approved the final manuscript.
